# Profiling the Impact of mGlu_7_/Elfn1 Protein Interactions on the Pharmacology of mGlu_7_ Allosteric Modulators

**DOI:** 10.1021/acschemneuro.5c00174

**Published:** 2025-07-21

**Authors:** Xia Lei, Zixiu Xiang, Alice L. Rodriguez, Margaret L. Wilson, Colleen M. Niswender

**Affiliations:** † Department of Pharmacology and Warren Center for Neuroscience Drug Discovery, 5718 Vanderbilt University School of Medicine, Nashville, Tennessee 37232, United States; ‡ Vanderbilt Institute of Chemical Biology, Vanderbilt University School of Medicine, Nashville, Tennessee 37232, United States; § Vanderbilt Brain Institute, Vanderbilt University School of Medicine, Nashville, Tennessee 37232, United States; ∥ Vanderbilt Kennedy Center, Vanderbilt University Medical Center, Nashville, Tennessee 37232, United States

**Keywords:** metabotropic glutamate, mGlu_7_, mGlu_8_, ELFN1, ELFN2, allosteric modulator, somatostatin, interneuron

## Abstract

The group III metabotropic glutamate receptors (mGlu receptors) are predominantly expressed presynaptically throughout the central nervous system (CNS) where they regulate the release of glutamate and GABA. These receptors have recently been shown to be anchored by transsynaptic expression of the laminin proteins ELFN1 and ELFN2. In particular, the mGlu_7_ receptor is localized at presynaptic active zones from pyramidal cells to somatostatin-containing interneurons with postsynaptic ELFN1, and this interaction drives the rapidly facilitating nature of these synapses in the hippocampus and cortex. Interestingly, individuals with mutations in *ELFN1* or *GRM7* genes present with attention-deficit hyperactivity disorder and epilepsy, and knockout mice of each of these proteins develop seizures with very similar time courses. In the current manuscript, we explore the hypothesis that the pharmacology of positive and negative allosteric modulators (PAMs and NAMs) of mGlu_7_ might be changed in the presence of ELFN1. These results showed that, across a range of NAMs, we observed similar efficacy in the presence of ELFN1. For PAMs, we observed decreased maximal potentiation when ELFN1 was present, but all examined compounds were still able to potentiate receptor signaling regardless of ELFN1 expression. Finally, we confirm that a tool PAM with mGlu_7_ activity is able to potentiate responses at pyramidal cell–somatostatin interneuron synapses where ELFN1 is expressed. These results suggest that, for the modulators shown here, native tissue activity should be retained in the presence of ELFN1 expression.

## Graphical abstract

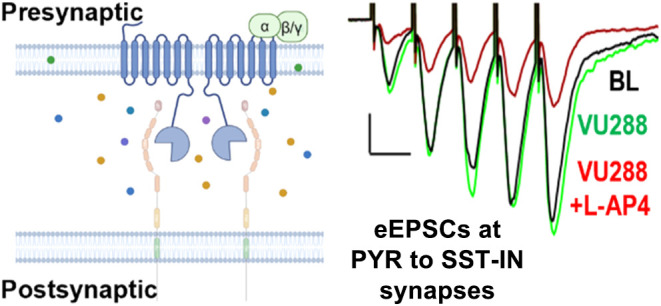

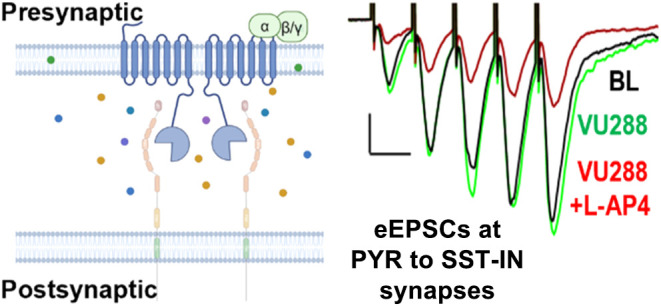

## Introduction

Metabotropic glutamate (mGlu) receptors are G-protein-coupled receptors (GPCRs) that modulate neurotransmitter release throughout the brain. mGlu receptors are categorized into three subfamilies based on sequence homology, G protein-coupling profile, and pharmacological regulation.^11^ The group III subfamily includes mGlu_4_, mGlu_6_, mGlu_7_, and mGlu_8_; with the exception of mGlu_6_, which is limited in expression to the retina,^12–14^ the other group III receptors are primarily localized to presynaptic terminals throughout the central nervous system (CNS) where they regulate the release of glutamate or GABA.^11^ Group III mGlu receptors offer attractive targets for therapeutics due to their modulatory role in synaptic function, allowing for potentially subtle shifts in signaling. Development of selective pharmacological tools for these receptors is anticipated to aid in characterizing their function in physiology and pathophysiology and validate their therapeutic potential.

Among the group III mGlu receptors, mGlu_7_ is unique in that it responds either constitutively^15,16^ or only to very high (high μM to mM,^17–20^ ) levels of glutamate and is specifically localized to the presynaptic active zones of glutamatergic and GABAergic synapses.^21–24^ Like the other group III mGlus, mGlu_7_ can heterodimerize with other receptor protomers in mGlu receptor groups II and III, which can dramatically change receptor pharmacology.^17,25–30^ Additionally, the group III mGlu receptors are transsynaptically “anchored” by the expression of laminin proteins termed Extracellular Leucine Rich Repeat and Fibronectin Type III Domain Containing 1 and 2 (ELFN1 and ELFN2, human; Elfn1 and Elfn2, rodent;^16,31–38^ schematic in Figure 1A). In the majority of cases, Elfns are expressed postsynaptically and the group III mGlu receptors are presynaptic; in the mouse retina, however, Cao et al. have shown that presynaptic Elfn1 anchors postsynaptically expressed mGlu_6_ in rod ON-bipolar cells, and *Elfn1* and *Grm6* knockout animals exhibit night blindness phenotypes due to synapse loss and an inability to properly connect rod photoreceptor cells.^35^ Therefore, interactions can occur in both directions between synapses to link group III mGlu receptors and Elfn proteins.

**Figure 1:**
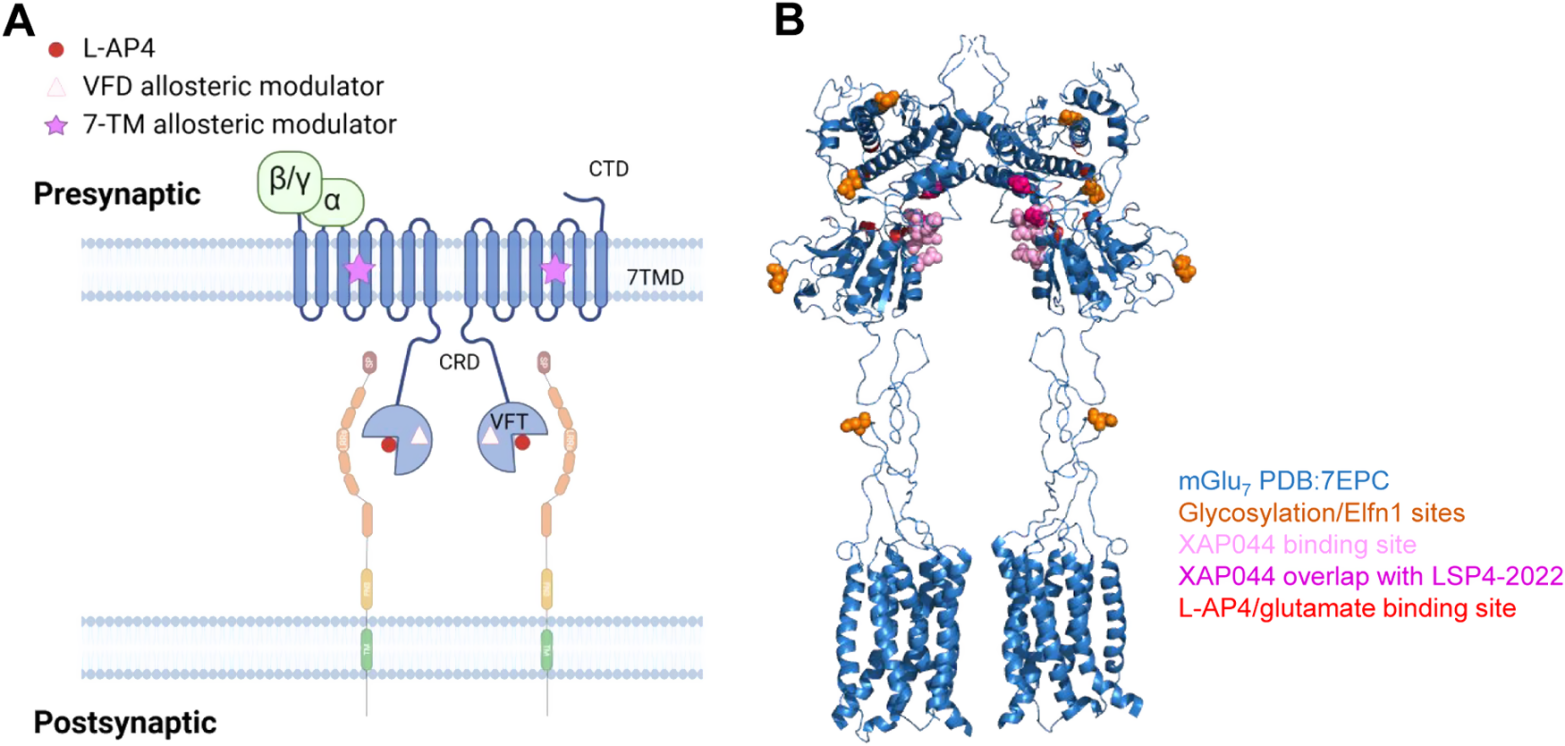
(A) Schematic of interactions between mGlu_7_ (blue) and Elfn1 (peach/green). Glutamate or the surrogate agonist L-AP4 is represented by red circles, Venus flytrap domain (VFD) allosteric modulator by pink triangles, and 7-transmembrane domain (7TMD) allosteric modulator by purple stars. (B) Mapping of relevant binding sites and glycosylation sites on mGlu_7_ (blue). Glycosylation/Elfn1 sites (orange), XAP044 binding sites (light pink), XAP044/LSP4-2022 binding site overlap (dark pink), and l-AP4/glutamate binding sites (red) are differentiated by color. Prepared using PyMOL Molecular Graphics System, Version 3.1 Schrödinger, LLC. Binding sites based on refs 1–10 .

Binding between group III mGlus and Elfns occurs via N-linked glycosylation sites in the ligand binding domain of the receptors (refs 8–10 ; positions of the relevant asparagines for mGlu_7_ are shown in Figure 1B, with Asn486 and Asn572 being most critical for mGlu_7_/Elfn1 binding). Knockout of either Elfn1 or Elfn2 in mice causes overlapping phenotypes such as seizures, susceptibility to convulsants, and hyperactivity.^32,37^ The interactions of mGlu_7_ with ELFN1 may be particularly important clinically, as mutations of each protein in humans and mice are correlated with attention-deficit hyperactivity disorder (ADHD) and seizures, and both *Elfn1* and *Grm7* knockout mice exhibit cognitive deficits, motoric changes, paradoxical decreases in locomotion induced by amphetamine, and seizures arising with the same developmental time course.^32,33,39–42^ Additionally, the *ELFN1* gene has recently been correlated with post-traumatic stress disorder (PTSD),^43,44^ and mGlu_7_ activity has been reported to be abnormal in patients with PTSD.^43^

Although interactions of Elfn1/2 and all of the group III mGlu receptors cause highly specific localization, in the case of mGlu_7_, one important location where an interaction has been described is at pyramidal cells (PYR) synapsing onto somatostatin-containing interneurons (SST-INs) in the mouse hippocampus and cortex.^16,31,33^ SST-INs exhibit dysfunction in numerous diseases such as epilepsy, attention-deficit hyperactivity disorder (ADHD), autism, and schizophrenia.^34,45–47^ In contrast, the mGlu_7_–Elfn1 interaction is not prominently seen in excitatory synapses onto other interneurons, such as those containing parvalbumin (PV-INs),^31^ suggesting that the specific expression of the mGlu_7_/Elfn1 complex in SST-INs can fine-tune precise synaptic responses to influence circuit-level control of hippocampal and cortical networks.^16^

As potentially druggable candidates, identification of new tools and drugs to target the group III receptors has become paramount, and we and others have had a long-standing interest in the development of small molecules targeting the mGlu_7_ receptor.^7,48–59^ As glutamate exhibits very low affinity for mGlu_7_, we and others often use surrogate agonists, such as l-AP4, in screening platforms to identify novel ligands that interact with the receptor. Recently, we have reported that glutamate and l-AP4 exhibit differences in activity in the presence of distinct allosteric modulators.^60^ Protein/protein interactions, including heterodimerization, of mGlu_7_ receptors may also alter pharmacological properties of ligands. This alteration may result in distinct pharmacology in tissue contexts in which the protein/protein interaction is present versus absent (e.g., refs 17,29 ). Therefore, an understanding of potential distinctions of mGlu_7_ ligand pharmacology is essential in the interpretation of pharmacological effects in native tissue and in vivo studies. In the current study, we tested the hypothesis that the activity of mGlu_7_ or group III mGlu receptor-targeted allosteric modulators would be different in the in vitro settings in which Elfn1 or Elfn2 was present versus those in which they were absent; in this report, we have focused on interactions of mGlu_7_ with Elfn1. Our data confirm that, as shown by Dunn et al. for mGlu_4_ and mGlu_6_,^36^ in vitro interaction of Elfn1 with mGlu_7_ reduces the efficacy of the orthosteric agonist, l-AP4, when Elfn1 is present compared to when the receptor is expressed alone. However, across the range of positive and negative allosteric modulators tested here, we find that these ligands are still able to potentiate and antagonize responses in the presence of Elfn1; for positive allosteric modulators (PAMs), however, potentiation was significantly reduced. More in-depth evaluation of one PAM, VU0422288, showed that potentiation was also observed in the presence of the endogenous agonist, glutamate, and potentiation induced by VU0422288 was present at cortical excitatory synapses onto SST-INs where Elfn1 is expressed. These data provide confidence that the compounds evaluated here will behave similarly in native tissue contexts, such as ex vivo slice electrophysiological preparations, regardless of the expression of Elfn1.

## Results and Discussion

### Activity of the mGlu_7_ Agonist l-AP4 Is Reduced in the Presence of Elfn1

We began our evaluation of mGlu_7_ ligands in the presence and absence of Elfn1 by using a coculture system as previously described^36,37^ with several modifications (Figure 2). Cells expressing rat mGlu_7_ and coexpressing G protein inwardly rectifying potassium (GIRK) channels 1 and 2 were cocultured with cells expressing either an empty pcDNA3 vector control or a plasmid containing Elfn1 in the pcDNA3 backbone. We then evaluated the activity of the orthosteric agonist l-AP4 and measured effects on maximal response and pEC_50_. All responses were normalized to the maximal effect of l-AP4 in the presence of the pcDNA3 control plasmid. These studies confirmed that, as previously shown for other group III mGlu receptors, the major effect of Elfn1 is a decrease in the maximal agonist response with a small decrease in pEC_50_ (refs 36,37 , Figure 3). Additionally, mGlu_7_ can interact constitutively with GIRK channels and Elfn1 increases the constitutive activities of mGlu_4_ and mGlu_6_.^36^ In our assay system, we did not observe differences in the baseline activity of mGlu_7_ in the presence versus absence of Elfn1 (shown in Figure 3A as overlapping baseline responses at low concentrations of l-AP4). This could be a consequence of receptor expression versus the studies performed in ref 36.

**Figure 2:**
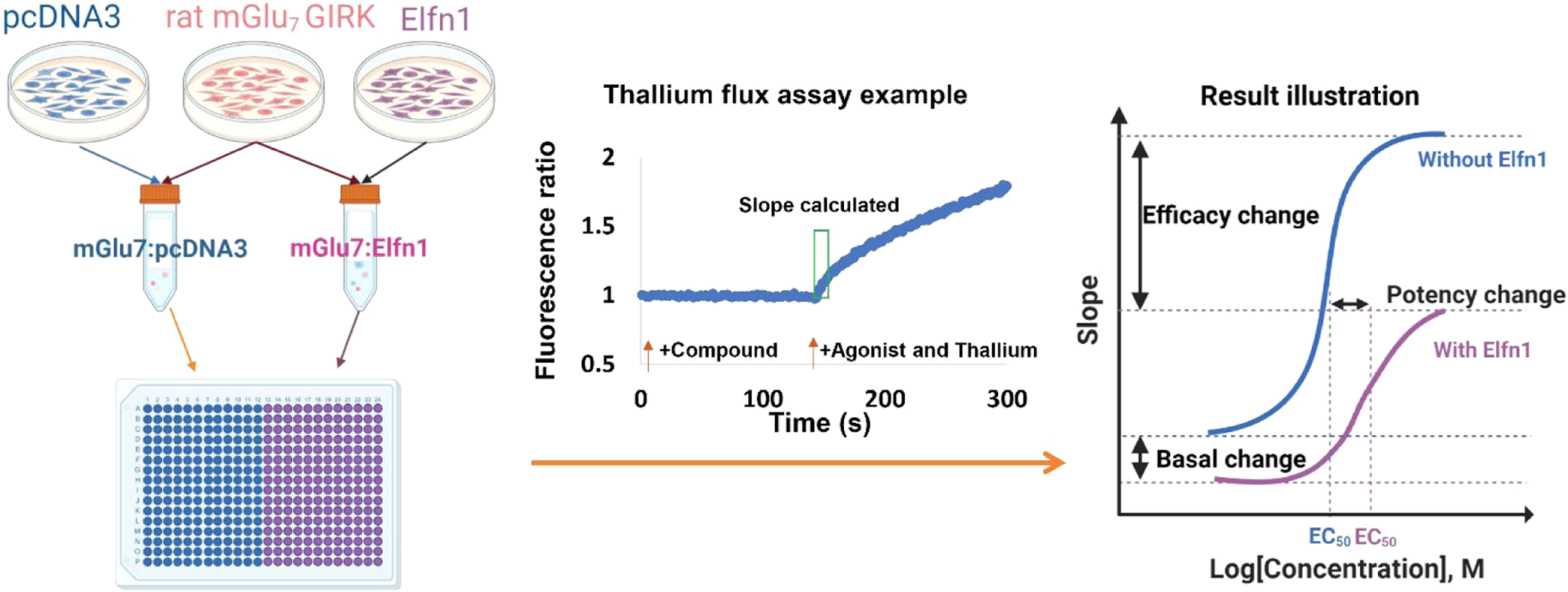
Assay design and workflow. (Left panel) Cells expressing rat mGlu_7_/GIRK (pink) were cocultured with pcDNA3 (blue) or Elfn1 (purple). (Middle panel) Activity was assessed using a thallium flux assay where the compound or vehicle was added at *t* = 1 s followed by the agonist at *t* = 141 s. The slope value for each kinetic trace was calculated for the time window of 145–155 s. (Right panel) The effect on the concentration–response curve in the presence (purple) or absence (blue) of Elfn1 can be seen by a change in efficacy, potency, and/or basal response. Note that while the decrease in basal response was reported for other mGlu receptors (Dunn et al., 2018), we did not observe this effect in the assay performed here.

**Figure 3:**
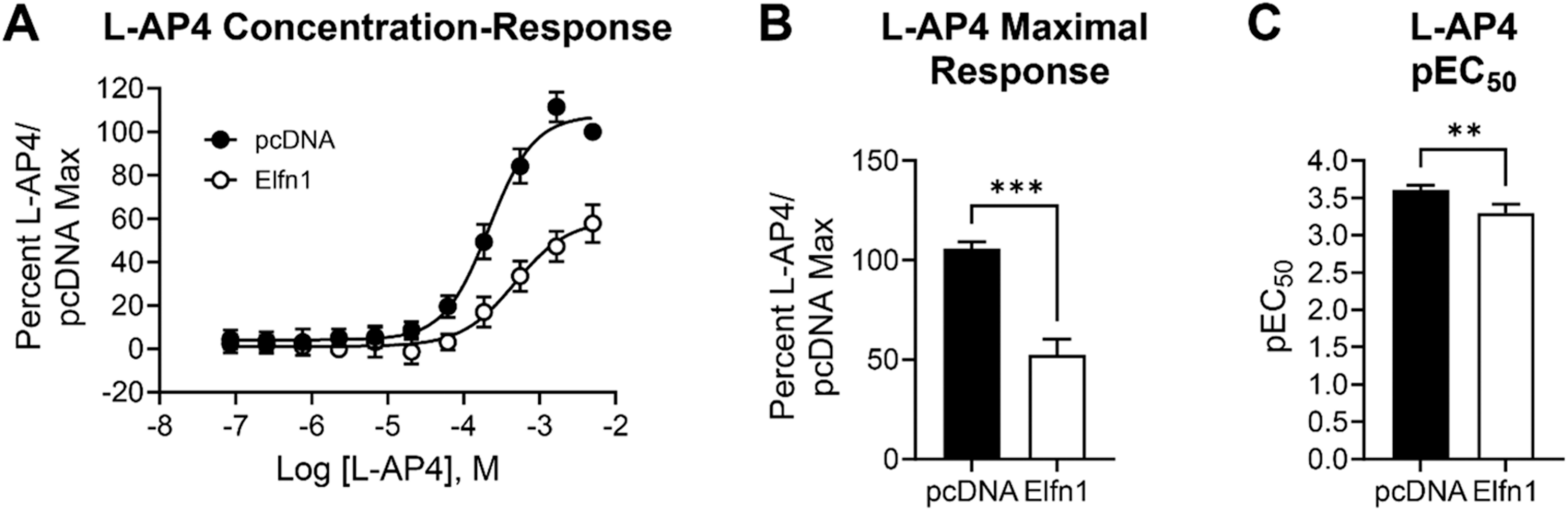
Coculture of mGlu_7_/GIRK-expressing HEK293 cells with HEK293 cells expressing Elfn1 results in a decreased maximal agonist response and a rightward shift in agonist potency. (A) Increasing concentrations of the mGlu_7_ agonist l-AP4 were applied to mGlu_7_/GIRK cells cocultured with cells expressing a pcDNA3 vector control (black symbols) or an Elfn1/pcDNA3 construct (white symbols) and thallium flux through GIRK channels was measured. (B) Maximal responses to l-AP4 were significantly reduced in the presence of Elfn1 (black versus white bars, ***p < 0.001, Students *t* test). (C) pEC_50_ of l-AP4 was significantly reduced in the presence of Elfn1 (**p < 0.01, Students *t* test). Data represent eight individual experiments performed in duplicate or triplicate and are shown as mean ± standard error of the mean (SEM).

### Structurally Diverse Antagonists and Negative Allosteric Modulators (NAMs) Show Similar Profiles in the Presence and Absence of Elfn1

To evaluate the activity of mGlu_7_ ligands in the presence and absence of Elfn1, we first chose three common antagonists with distinct properties: the orthosteric antagonist LY341495,^20^ the negative allosteric modulator (NAM) ADX71743,^56^ which is predicted to bind in the transmembrane domain of mGlu_7_, and the compound XAP044, an antagonist that binds in the extracellular Venus Flytrap Domain of mGlu_7_ at a site that is distinct from the l-AP4/glutamate site but overlaps with an agonist called LSP2-4022 (refs 5–7 ; binding sites mapped onto the structure of mGlu_7_ in Figure 1B). Structures of these compounds are shown in Figure 4. We first tested the effect of a single concentration of antagonist or NAM (10 μM) on an l-AP4 concentration–response curve (CRC) with or without Elfn1 coseeding, and the results are shown in Figure 5A,E,I. We calculated the baseline activity (the average response observed at the bottom three l-AP4 concentrations of each curve; Figure S1; Table 1) and the maximal activity (the average response observed at the top two l-AP4 concentrations of each curve; Figure 5B,F,J; Table 1). These results confirmed that baseline mGlu_7_/GIRK responses were not significantly different in the presence of Elfn1 (red versus black bars in Figure S1). However, XAP044 and, more prominently, ADX71743, induced responses that were below the baseline response in the presence of inactive concentrations of l-AP4, indicative of inverse agonist activity; this effect was observed regardless of the presence of Elfn1 (blue and green bars in Figure S1). When we compared the maximal responses of each compound in the presence and absence of Elfn1, we observed that each compound inhibited the response regardless of the presence of Elfn1, and responses were not significantly different between the minus and plus Elfn1 conditions (blue versus green bars, Figure 5B,F,J, Table 1). Determination of the percentage of blockade in the presence and absence of Elfn1 (Figure 5C,G,K, Table 1) showed that the orthosteric antagonist LY341495 inhibited the response to a similar degree regardless of Elfn1. The NAMs XAP044 and ADX71743, however, induced significantly better inhibition in the presence of Elfn1 when tested at a 10 μM response. To better evaluate effects across a range of antagonist/NAM concentrations, we performed full concentration–response evaluation for each compound at a 600 μM concentration (a concentration that induces an 80% maximal response) of l-AP4 and responses were normalized to account for the lower maximal agonist response observed in the presence of Elfn1 (Figure 5D,H,L). These studies revealed no significant effect on the pEC_50_ of each compound or on the maximal inhibition induced at a 30 μM concentration (Table 1), suggesting that, although the agonist response is reduced, the antagonists or NAMs block agonist-mediated activity to a similar degree regardless of the presence of Elfn1 when tested across a full concentration range with an EC_80_ concentration of l-AP4.

**Figure 4:**
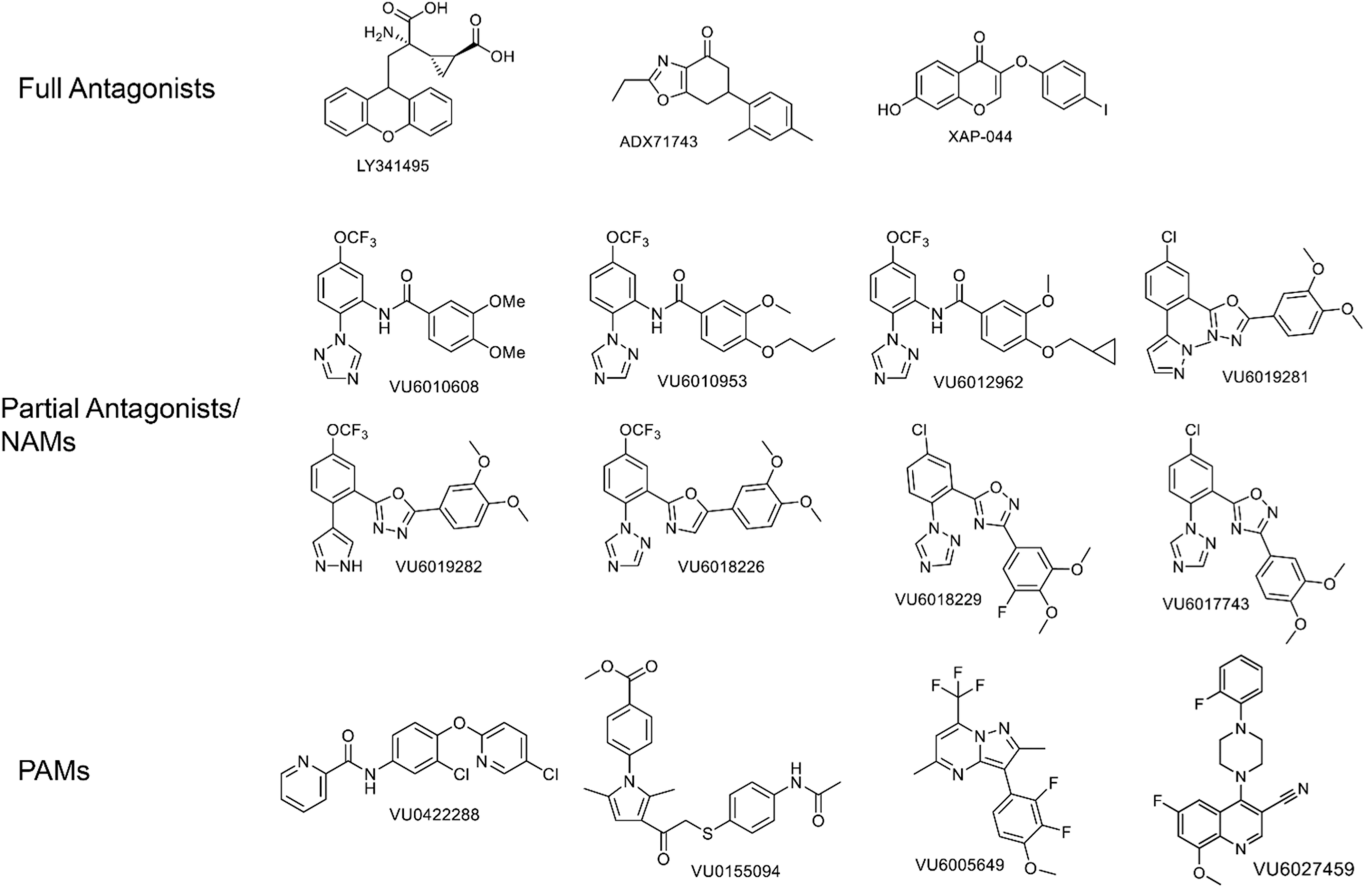
Compound structures.

**Figure 5:**
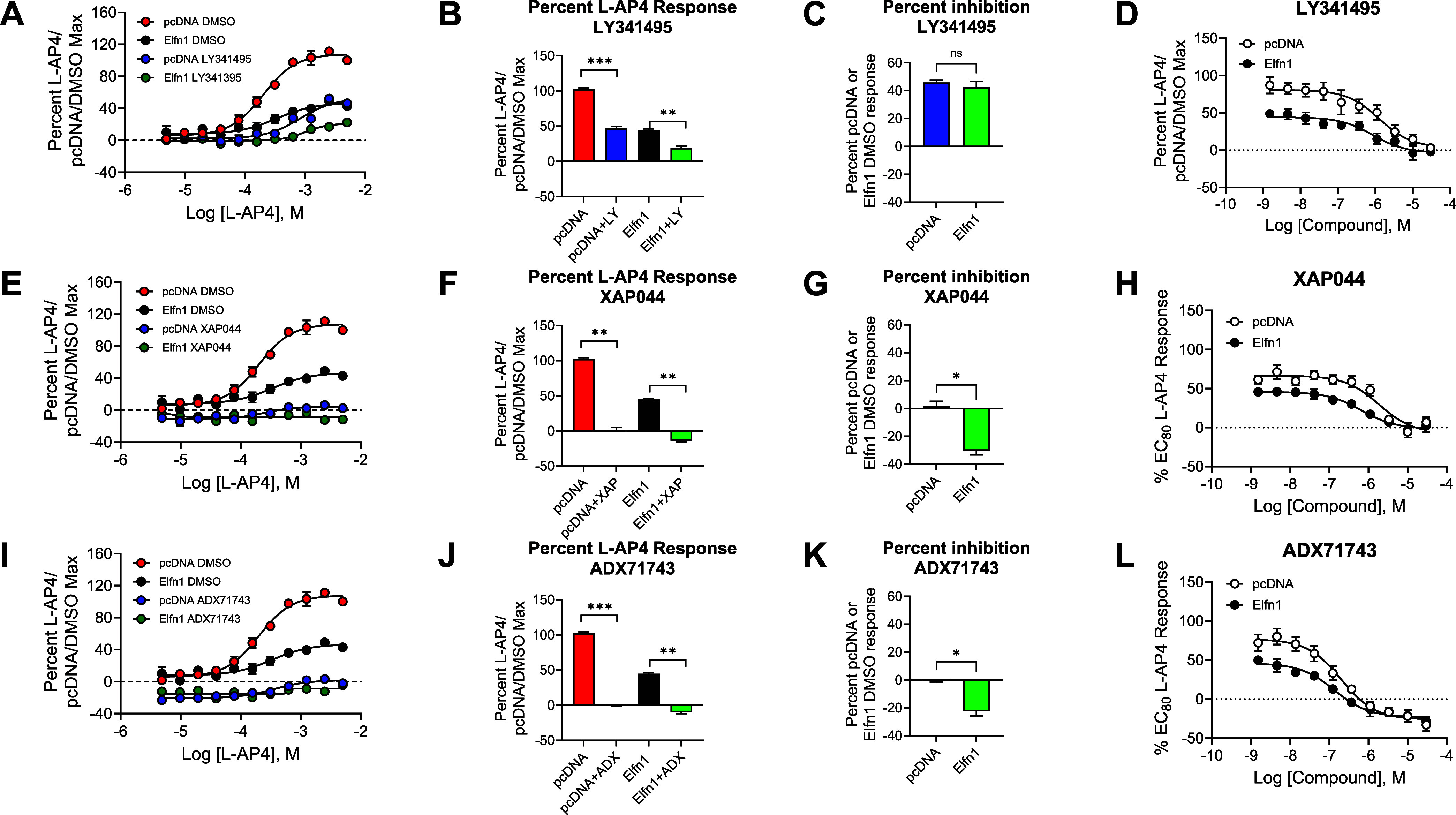
Three distinct antagonists that interact with different domains on the mGlu_7_ receptor show similar profiles in the absence and presence of Elfn1. Top row = orthosteric antagonist, LY341495; middle row = Venus flytrap domain-interacting antagonist, XAP044; bottom row = transmembrane domain negative allosteric modulator, ADX71743. Red = pcDNA + dimethyl sulfoxide (DMSO), black = Elfn1 + DMSO, blue = pcDNA + antagonist, green = Elfn1 + antagonist. DMSO vehicle or 10 μM antagonist was added to mGlu_7_/GIRK cells cocultured with cells expressing a pcDNA3 vector control or an Elfn1/pcDNA3 construct. Increasing concentrations of l-AP4 were added and thallium flux through GIRK channels was measured. (A, E, I) Full concentration–response curves. (B, F, J) Maximal responses were quantified by averaging the responses of the top two concentrations of l-AP4. Paired *t* test between vehicle and antagonist conditions, *p < 0.05; **p < 0.01; ****p < 0.0001; ns = not significant. (C, G, K) Percent inhibition between the pcDNA (blue) and Elfn1 (green) conditions were measured. Paired *t* test between conditions, *p < 0.05, ns = not significant. (D, H, L) Full concentration–response curves of antagonists were performed in the presence of 600 μM l-AP4, and responses were normalized to the response of 600 μM l-AP4 in the presence of pcDNA3 alone. Data represent three experiments performed in duplicate or triplicate and are shown as mean ± SEM.

**Table 1:** Baseline, Maximal, % Inhibition, pIC_50_, and Minimal Curve Fit Responses for LY341495, ADX71743, and XAP044 in the Presence and Absence of Elfn1^a^

	baseline Resp ± SEM		Maximal Resp ± SEM		% inhibition Resp ± SEM		compound pIC_50_ ± SEM		compound Min Resp ± SEM	
	pcDNA	Elfn1	pcDNA	Elfn1	pcDNA	Elfn1	pcDNA	Elfn1	pcDNA	Elfn1
DMSO	6.8 ± 2.3	8.5 ± 2.8	102.6 ± 1.8	44.9 ± 1.3	NA	NA	NA	NA	NA	NA
LY341795	8.4 ± 2.8	1.8 ± 3.4^#^	47.1 ± 4.6***	19.2 ± 2.4^##^	45.9 ± 1.8	42.5 ± 4.1	5.87 ± 0.21	6.00 ± 0.11	3.3 ± 1.7	–2.1 ± 2.6
ADX71743	–21.5 ± 2.0***	–12.7 ± 2.9^#^	–0.08 ± 1.4***	–10.1 ± 1.7^##^	–0.11 ± 1.4	–22.4 ± 3.4*	6.71 ± 0.08	6.85 ± 0.01	–32.9 ± 8.0	–28.5 ± 5.5
XAP044	–11.4 ± 4.8*	–6.5 ± 1.3^#^	1.7 ± 3.6**	–13.2 ± 1.7^##^	1.6 ± 3.5	–30.4 ± 2.9*	5.78 ± 0.08	6.42 ± 0.20	6.9 ± 6.5	2.5 ± 8.4

a Data were compared with paired *t* tests and represent the mean ± SEM of three independent experiments performed in duplicate or triplicate. Response of the compound between the DMSO and NAM conditions for either pcDNA (*) or Elfn1 (#) were compared. * ^or^^#^ *p* < 0.05, ** ^or^^##^ *p* < 0.01, ***p < 0.001. For % inhibition, the comparison was between pcDNA and Elfn1 for each compound (*). The table refers to data in Figures S1 and 5 (baseline, Figure S1; maximal response, Figure 5B,F,J; pEC_50_, percent inhibition at 10 μM, Figure 5C,G,K; minimal response, Figure 5D,H,L).

### Structure–Activity Relationships of a Series of mGlu_7_ NAMs are Maintained in the Presence of Elfn1

We have recently reported on a series of mGlu_7_ NAMs that exhibit a range of efficacies at mGlu_7_, including some that induce complete blockade and other structurally related compounds that only partially block l-AP4 responses in a saturable manner (ref 50, Figure 4).

These compounds were tested at a 10 μM concentration in the presence of a complete concentration–response curve for l-AP4 (Figure 6); effects on basal and maximal responses are shown in the Supporting Figures. As shown in Figures S2 and S3 and Table 2, compounds significantly decreased baseline responses in mGlu_7_ cells cocultured with pcDNA3 (red versus blue bars), and several also showed this behavior when Elfn1 was present (for VU6018226 and VU6019282, these responses did not reach significance versus controls for the Elfn1 condition). When maximal responses were compared, all NAMs were active in the absence or presence of Elfn1 (Figures 6 and S3 and Table 2). When the responses of all NAMs were converted to percent inhibition and plotted for activity in the presence of pcDNA3 versus Elfn1, a strong positive correlation (*R* ^2^ = 0.88, ****p < 0.0001) was observed (Figure 7) for all of the NAMs tested in Figures 5 and 6. This suggests that all of these NAMs interact with and antagonize the receptor to a similar relative degree regardless of the presence of Elfn1, despite the reduced maximal response induced by the l-AP4 agonist when Elfn1 is present.

**Figure 6:**
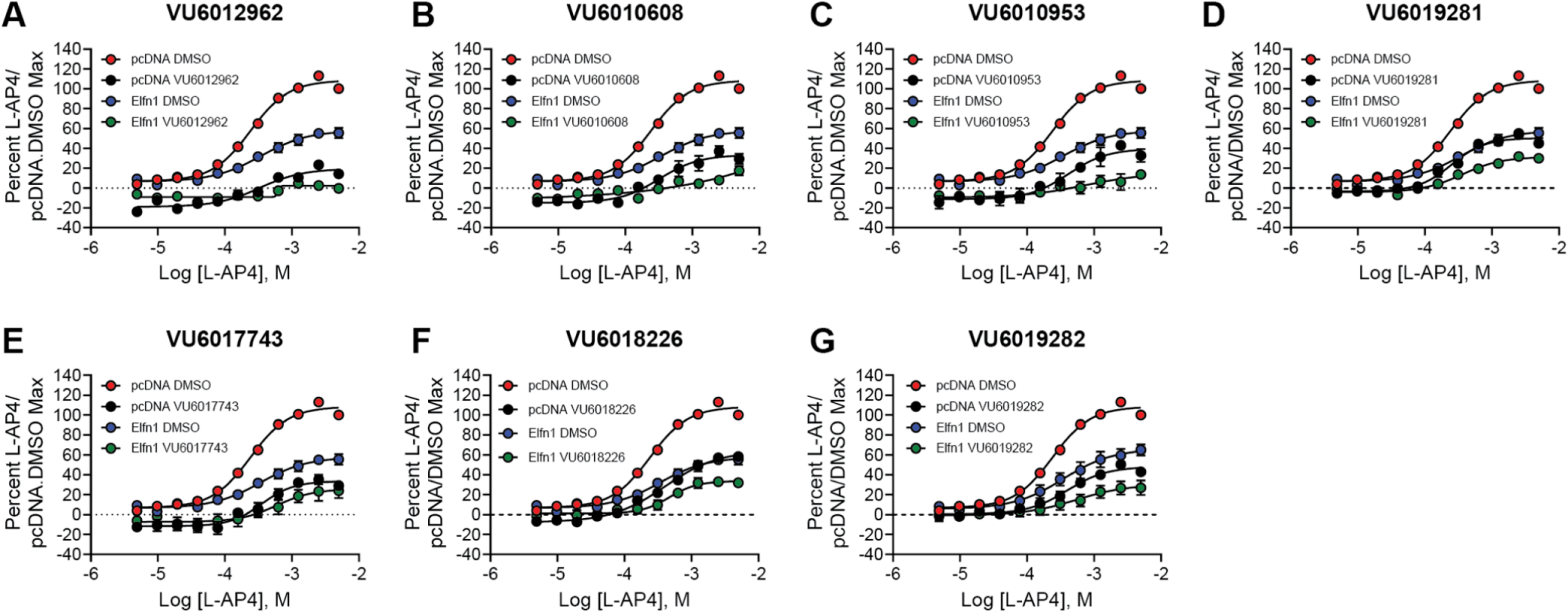
Structure–activity relationships of a range of mGlu_7_ NAMs are maintained in the presence of Elfn1. Red = pcDNA + DMSO, black = pcDNA + antagonist, blue = Elfn1 + DMSO, green = Elfn1 + antagonist. DMSO vehicle or 10 μM antagonist was added to mGlu_7_/GIRK cells cocultured with cells expressing a pcDNA3 vector control or an Elfn1/pcDNA3 construct. Increasing concentrations of l-AP4 were added, and thallium flux through GIRK channels was measured for a range of NAMs. Data represent three experiments performed in duplicate or triplicate and are shown as mean ± SEM.

**Figure 7:**
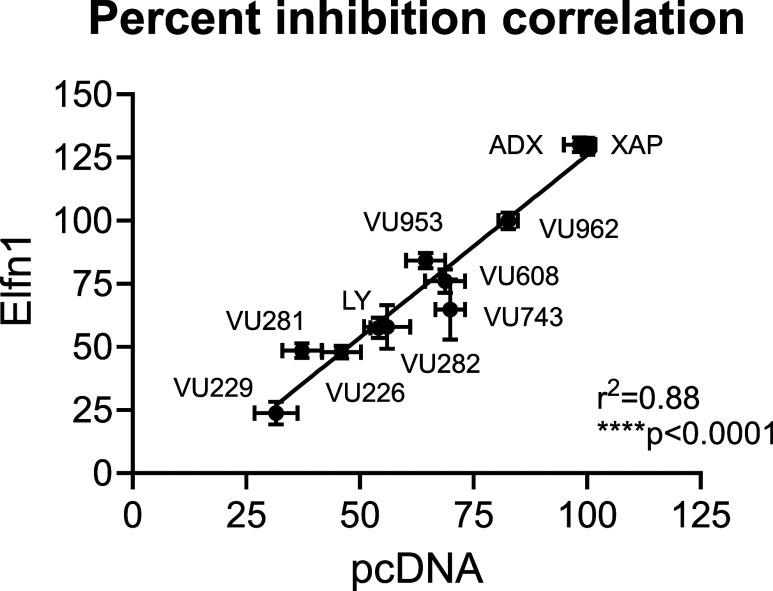
Across all compounds, there is a strong correlation in blocking mGlu_7_-mediated response in the absence or presence of Elfn1. The maximal responses of all NAMs examined in Figures 5 and 6 were plotted for activity in the presence of pcDNA3 (*x*-axis) or Elfn1 (*y*-axis) and a linear regression was performed. *r* ^2^ = 0.88, ****p < 0.0001.

**Table 2:** Baseline and Maximal Responses for a Series of mGlu_7_ NAMs with a Range of Efficacies in the Presence and Absence of Elfn1^a^

	baseline response ± SEM		l-AP4 pEC_50_ ± SEM		maximal response ± SEM	
	pcDNA	Elfn1	pcDNA	Elfn1	pcDNA	Elfn1
DMSO	7.4 ± 1.1	7.8 ± 1.9	3.71 ± 0.03	3.49 ± 0.07	105.2 ± 1.5	54.8 ± 4.7
VU6012962	–19.1 ± 2.4****	–8.1 ± 2.0**	3.48 ± 0.04*	N/A	32.0 ± 4.4****	10.8 ± 2.4***
VU6010608	–14.5 ± 2.6****	–9.0 ± 0.9***	3.43 ± 0.07*	N/A	36.4 ± 4.3****	7.0 ± 1.2***
VU6010953	–12.5 ± 2.2****	–8.1 ± 1.1***	3.38 ± 0.05**	N/A	17.9 ± 2.4****	0.0 ± 1.4****
VU6019281	–3.3 ± 1.7***	–3.1 ± 2.6**	3.57 ± 0.04	3.39 ± 0.11	49.5 ± 3.5****	30.9 ± 2.3***
VU6017743	–11.5 ± 4.7***	–5.7 ± 5.9*	3.45 ± 0.05*	3.22 ± 0.04	32.2 ± 3.3****	23.7 ± 8.4**
VU6018226	–6.6 ± 1.8****	0.2 ± 3.5	3.39 ± 0.05*	3.33 ± 0.03	47.0 ± 5.1****	26.9 ± 6.9**
VU6019282	–5.3 ± 3.7**	–0.7 ± 4.5	3.37 ± 0.05**	3.16 ± 0.15	57.8 ± 3.9****	32.5 ± 3.8**

a Data were compared with paired *t* tests and represent the mean ± SEM for three independent experiments performed in duplicate or triplicate. *t* tests compare the response of the compound between the DMSO and each NAM condition. *p < 0.05, *p < 0.01, ***p < 0.001, ****p < 0.0001. Table refers to data in Figure 6.

### PAMs Also Exhibit Similar Profiles in the Presence or Absence of Elfn1

We next turned to a series of structurally diverse PAMs of mGlu_7_ (Figure 4) and performed similar concentration–response curves of l-AP4 in the presence of 10 μM of each PAM plus and minus coculture with Elfn1 (Figure 8 and Table S1). As before, responses were normalized to the percent l-AP4 response in the presence of pcDNA and the DMSO vehicle. Of the PAMs tested, only VU6005649 resulted in an increase in the baseline response, which reflects the allosteric agonist activity of the compound that we have previously reported (Figure 8A, ref 53). When maximal responses were measured (Figure S4), we observed significant potentiation in the absence or presence of Elfn1 for all PAMs. As VU6005649 exhibits agonist activity in addition to PAM activity, we also compared the percent agonist activity of VU6005649 after normalization to either the pcDNA3 or Elfn1 conditions. Agonist activity was found to be reduced in the presence of Elfn1 when compared to the pcDNA3 control (Figure S4E). We then performed concentration–response experiments for each PAM using a concentration of l-AP4 that elicits a 20% maximal response in each cell line (Figure 9A–D and Table S2). When expressed as fold potentiation, compounds all induced at least a 2-fold potentiation of the response in the presence of Elfn1, although responses were significantly lower in the presence of Elfn1 when compared to the fold potentiation induced in the presence of pcDNA3 (Figure 9E).

**Figure 8:**
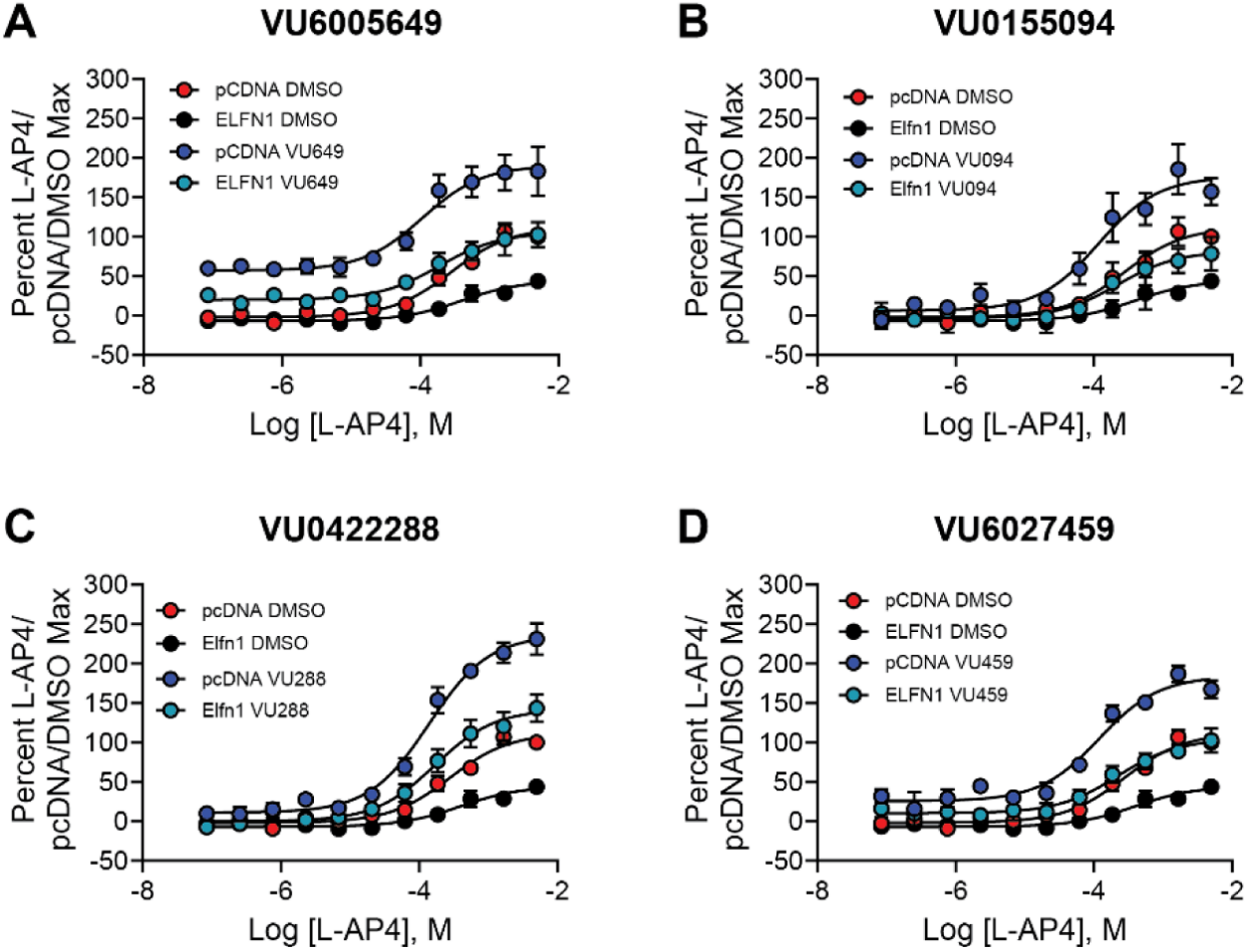
All examined PAMs potentiate mGlu_7_ responses in the presence or absence of Elfn1. Red = pcDNA3 + DMSO, blue = pcDNA3 + PAM, black = Elfn1 + DMSO, green = Elfn1 + PAM. DMSO vehicle or 10 μM PAM was added to mGlu_7_/GIRK cells cocultured with cells expressing a pcDNA3 vector control or an Elfn1/pcDNA3 construct. Increasing concentrations of l-AP4 were added and thallium flux through GIRK channels was measured. Data represent three experiments performed in duplicate or triplicate and are shown as mean ± SEM.

**Figure 9:**
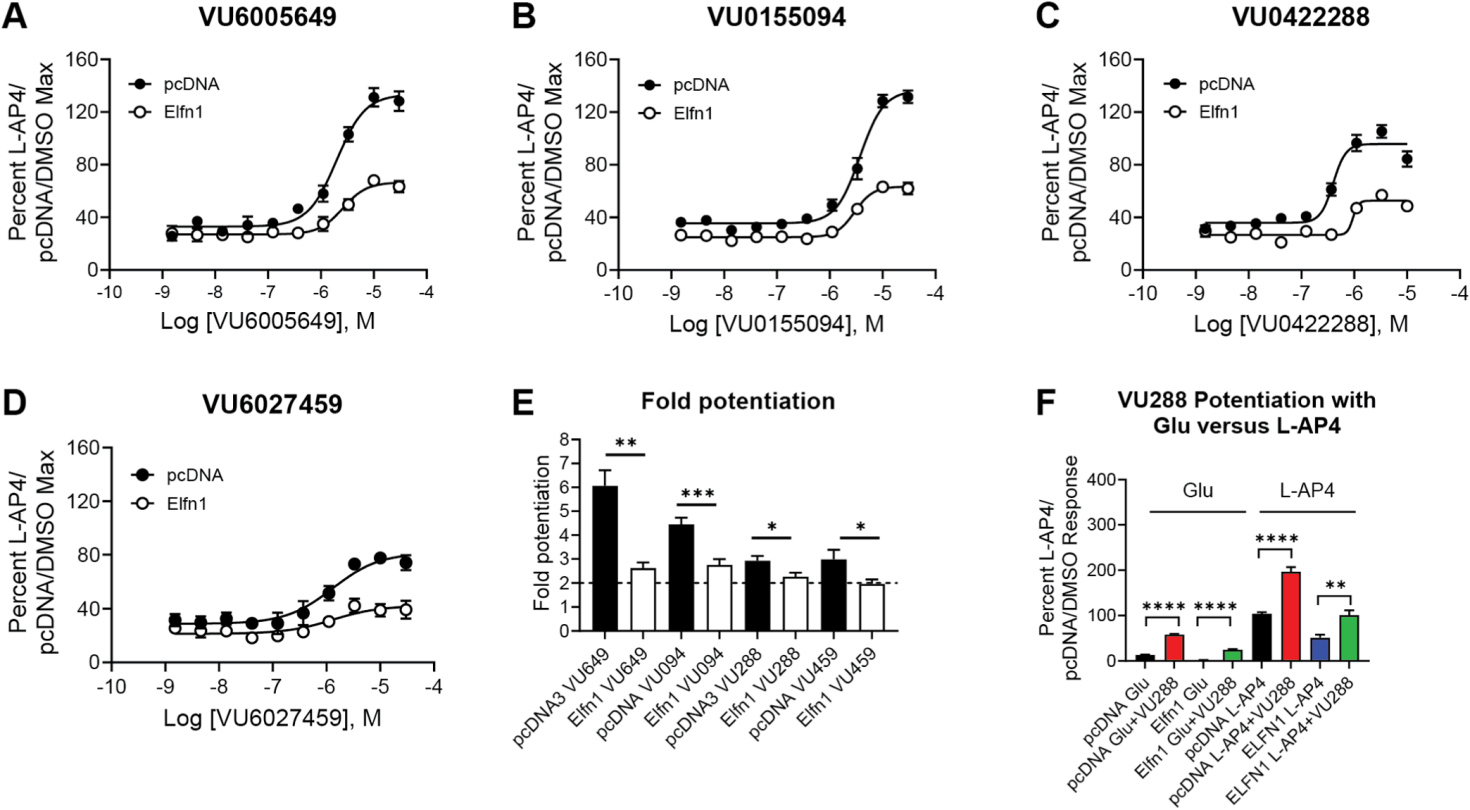
All tested PAMs potentiate EC_20_ l-AP4 responses in the presence of Elfn1 and VU0422288 is active with glutamate. Increasing concentrations of (A) VU6005649, (B) VU0155094, (C) VU0422288, and (D) VU6027459 were added to mGlu_7_/GIRK cell cocultures with either pcDNA3 (black) or Elfn1 (white) and thallium flux was measured. (E) Fold potentiation of an EC_20_ concentration of l-AP4 was calculated, showing that all PAMs exhibited lower maximal levels of potentiation when Elfn1 is present. Unpaired *t* tests between vehicle and PAM conditions, *p < 0.05, **p < 0.01 between the pcDNA (black) and Elfn1 (white) conditions. (F) VU0422288 potentiates glutamate responses in the presence of Elfn1. **p < 0.01, ****p < 0.0001. Data represent three experiments performed in duplicate or triplicate and are shown as mean ± SEM.

The agonist l-AP4 is often used as a surrogate for glutamate at mGlu_7_ due to the low affinity and activity of glutamate at this receptor.^11,17,20^ However, allosteric modulators can exhibit a phenomenon called “probe dependence”, in which their activity differs depending on the agonist used or based on other protein interactions with a receptor.^61,62^ We have shown that, for mGlu_7_, glutamate and l-AP4 appear to activate the receptor differently, which impacts allosteric modulator pharmacology.^60^ As shown in Figure 9F, we examined the activity of one PAM we have used in tissue slices and in vivo, VU0422288,^55,63^ in the presence of either l-AP4 or glutamate. These studies showed that VU0422288 was able to significantly potentiate responses of mGlu_7_ with both agonists in the presence or absence of Elfn1 when glutamate was used as the agonist.

### VU0422288 is Active at Pyramidal Cell to SST-INs, Indicating PAM Activity at a Synapse at which Elfn1 is Expressed

Elfn1 is highly expressed in SST-INs in the hippocampus and cortex and has been shown to interact specifically with mGlu_7_ to regulate glutamate release.^16,31,33^ Based on our in vitro data showing that the PAM VU0422288 can potentiate mGlu_7_ responses in either the absence or presence of Elfn1, we tested the hypothesis that VU0422288 would potentiate evoked excitatory postsynaptic currents (eEPSCs) at synapses projecting from local pyramidal cells to SST-INs in the prefrontal cortex (PFC). Shown in Figure 10A are representative traces of eEPSCs recorded from SST-INs in response to 5-pulse stimulation with baseline in black, illustrating the characteristic facilitating nature of pyramidal cell (PYR) to SST-IN synapses.^16^ Application of 30 μM l-AP4 reduced eEPSC amplitude (Figure 10A,B, left), as previously reported.^16^ In contrast to the effect of the agonist l-AP4, application of 10 μM PAM VU0422288 alone induced no change in eEPSC amplitude, compared to the baseline (green versus black, Figure 10A, right and Figure 10B, middle). However, application of a combination of 30 μM l-AP4 and 10 μM VU0422288, compared to l-AP4 alone, induced further depression of eEPSCs (Figure 10A, right, Figure 10C), indicating that VU0422288 is active at potentiating responses at these synapses where Elfn1 is expressed. In addition, analysis of 5-pulse-evoked short-term synaptic plasticity revealed that VU0422288 had a significant effect on synaptic facilitation at these synapses (Figure S5), suggesting that the reduction of eEPSC1 amplitude in the presence of the combination of VU0422288 and l-AP4 is due to a decrease in release probability, a presynaptic mechanism of the action.

**Figure 10:**
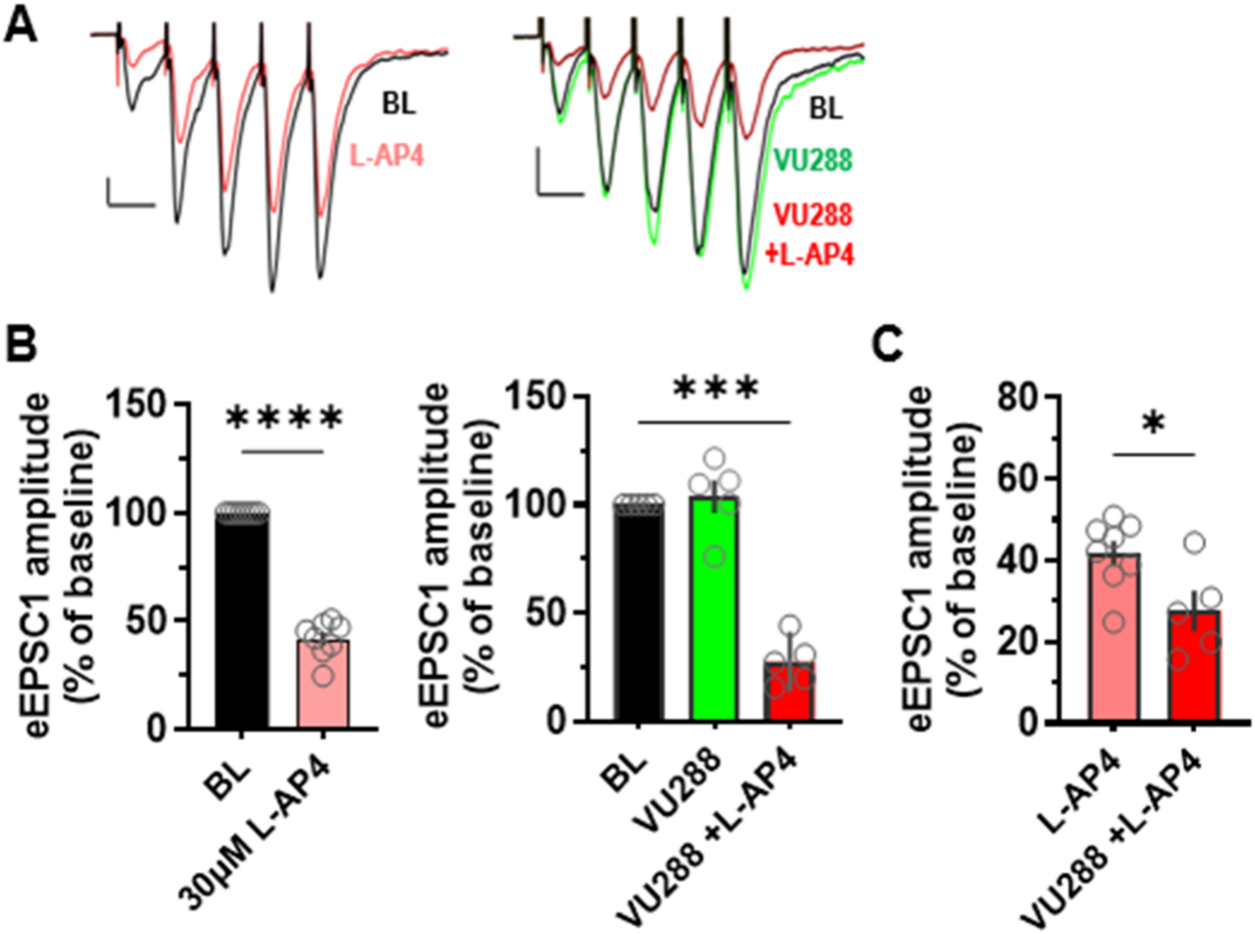
The PAM VU0422288 is active in potentiating responses at SST-INs. (A) Left, Example of averaged traces of 5-pulse-evoked EPSCs recorded from SST-INs in the PFC during baseline (BL, black) and application of 30 μM l-AP4 (light red); and right, during baseline (black), 10 μM VU0422288 (VU288, green), and a combination of VU0422288 and 30 μM l-AP4 (dark red). Scale bars: 50 pA/20 ms. (B) Bar graphs summarizing the normalized amplitude of EPSC_1_ in baseline and l-AP4 conditions (left, ****p < 0.0001, paired *t* test, *n* = 8), and in baseline, VU288, and a combination of VU0422288 and l-AP4 conditions (right). Repeated measures (RM) one-way analysis of variance (ANOVA), *F*(1.572, 6.288) = 73.09, ****p < 0.0001, with post hoc Dunnett’s test; middle, ***p < 0.0005, *n* = 5. (C) Summary of percentage change of EPSC_1_ amplitude during application of 30 μM l-AP4 in the absence and presence of VU0422288 (*p < 0.05, unpaired *t* test; *n* = 8 and 5, respectively).

### Consequences of Elfn/Group III mGlu Receptor Interactions

It has recently been recognized that the group III mGlu receptors can interact with proteins called Extracellular Leucine Rich Repeat and Fibronectin Type III Domain Containing 1 and 2 (ELFN1 (human)/Elfn1 (rodent) and ELFN2/Elfn2)^16,33–35^ that are expressed on the opposite side of the synapse. In recent years, the complex regulation of GPCRs has been expanded to include cellular interacting proteins such as ELFNs as well as other single-transmembrane proteins such as the neurexins, teneurin, the fibronectin domain-containing proteins Flrt1–3, contactins, and neuroligins (reviewed in refs 34,64 ). In addition, latrophilin receptors, such as the adhesion GPCR subfamily, interact with Flrts and teneurins to control the type and specificity of synaptic connections between neurons (reviewed in ref 64).

The two ELFN proteins exhibit differences in expression in various neuronal populations and can be expressed either pre- or postsynaptically depending on the cell type.^32,33,35,37,65^ The ELFNs interact with all of the group III mGlu receptors but not mGlu receptors in groups I and II,^36–38^ demonstrating selectivity of this protein/protein-mediated regulation. In the mouse retina, Elfn1 is expressed presynaptically in rod cells and binds to mGlu_6_ expressed on postsynaptic rod ON-bipolar cells. Elfn2 binds to mGlu_6_ in cone ON-bipolar cells,^35,38^ and the two ELFN proteins work in concert to control appropriate retinal wiring. In many neuronal locations, the ELFNs are expressed postsynaptically, positioned to “anchor” the presynaptic group III receptors in place. This may be most important for mGlu_7_, which exhibits high concentration within the presynaptic active zone,^22,24^ particularly in pyramidal cells synapsing onto mGlu_1_- and somatostatin-expressing interneurons.^24^ Knockout of Elfn1 results in mislocalization of mGlu_7_ protein in the presynaptic terminal away from the active zone.^31^ Additionally, knockout of Elfn2 in mice results in decreased protein expression of all of the group III mGlu receptors in the brain,^37^ indicating that mGlu/ELFN interactions are important for both stabilizing group III mGlu protein expression and localization.

Binding of the ELFNs to group III mGlu receptors occurs through N-linked glycosylation sites that are specific to the four group III mGlu receptors (mGlu_4,6,7,8_).^8,9^ For mGlu_6_, these glycosylation sites are Asn290, 445, 473, and 561, with mutation of Asn445 showing the greatest effect on ELFN1 and 2 binding.^9^ Glycosylation to mature forms of glycans is required for binding interaction.^9^ Notably, mutations in the ligand binding domain of mGlu_6_ that are found in patients with congenital night blindness also affect maturation of glycosylation and shunt mGlu_6_ through an alternate trafficking pathway outside of the Golgi, inhibiting the integration of mature N-glycans and preventing interaction with ELFN1 proteins.^10^ Miller et al. showed that, if these identified glycosylation sites for mGlu_6_ are mapped onto the corresponding mGlu_4_ structure, the glycosylated amino acids are more than 20 Å removed from the glutamate binding site.^9^ For mGlu_7_, the two most important interaction sites have been shown to be Asn486 and Asn572,^8^ which are also spatially distinct from the orthosteric binding pocket (Figure 1B). Additionally, they are predicted to be some distance away from the binding site of the NAM XAP044, which interacts in the Venus flytrap domain at a site distinct from the glutamate binding site.^6,7^ It has also recently been shown that the ELFNs function as dimers in the cell, and heterodimerization between the two proteins can occur.^66^ Coupled with the ability of the mGlus to heterodimerize, this results in numerous protein/protein complex combinations between the ELFNs and mGlu receptors.

Interestingly, knockout of each of the mouse *Elfn* homologues causes similar phenotypes, including seizures and hyperactivity.^32,33,37^ Genomic-linkage and primary mutation studies have established significant correlations between *GRM7* and *ELFN1* mutations with numerous neurological disorders, including ADHD and response to ADHD medications, post-traumatic stress disorder, epilepsy, schizophrenia, and idiopathic autism.^32,33,67–82^ The above findings in humans suggest that mutations in either *GRM7* or *ELFN1* are deleterious. *Grm7* ^*–/–*^ mice exhibit seizures and cognitive impairments, and mutations in *GRM7* in humans cause severe neurodevelopmental phenotypes.^39–42,83–87^ Additionally, *Grm7* ^*–/–*^ and *Elfn1* ^*–/–*^ mice also exhibit a reduced locomotor and EEG responses to amphetamine^32,40^ and mutations in *ELFN1* have been linked to ADHD in humans and mice.^32,33,40^ These findings suggest that there is an intimate and important relationship between mGlu_7_ and ELFN1.

In rodents, mGlu_7_ and Elfn1 interact between pyramidal cells (PYRs) synapsing onto SST-INs in both the hippocampus and cortex.^16,31^ These neurons act as feedback circuits to increase inhibition of PYR cells when PYRs become overactive. As shown in Figure 10, PYR → SST-IN synaptic responses facilitate upon successive stimulations, which progressively increases the excitatory drive to GABAergic SST-INs. This results in feedback inhibition to PYRs, and this facilitation of synaptic drive is specific to SST-INs versus other interneuron cell types.^16,33^ Interestingly, increasing expression of Elfn1 in parvalbumin interneurons (PV-INs) causes them to shift their firing pattern from rapidly depressing to facilitating.^31^ In particular, Stachniak et al. have shown that the interaction of Elfn1 with mGlu_7_ suppresses initial glutamate release in response to high-frequency activity, creating short-term synaptic facilitation of excitatory currents upon additional action potentials.^16^ In *Elfn1* ^*–/–*^ mice, the initial release probability is increased, while the multipulse facilitation is diminished.^16^ Initially, high glutamate release onto SST-INs would be expected to acutely increase SST-IN-mediated inhibition of PYRs; however, the decreased glutamate release predicted during the later trains (representative of higher neuronal activity) decreases glutamate release from PYRs onto SST-INs, preventing appropriate inhibition of PYR output under periods of intense stimulation. Dysfunction of SST-INs has been proposed as a mechanism underlying ADHD, schizophrenia, and epilepsy,^47,88–91^ in which the specific loss of SST-IN function is observed compared to other interneuron subtypes.^92^ Together, these findings are consistent with a critical role for the mGlu_7_/Elfn1 complex in regulating the activity of SST-INs within larger neuronal circuits, and mGlu_7_/Elfn1 synapses between PYR and SST-INs may represent a point of intersection for the overlapping neuropathological phenotypes seen in human and rodent populations that have loss-of-function mutations in either mGlu_7_ or Elfn1 proteins.

Here, we probed the mGlu_7_/Elfn1 interaction using a variety of mGlu_7_ modulators to test the hypothesis that their pharmacology could be unique in the presence versus absence of Elfn1. These studies confirmed that the response of mGlu_7_ to the group III mGlu receptor agonist, l-AP4, is significantly reduced in the presence of Elfn1, which corroborates results reported in ref 36 for other group III mGlu receptors. We next turned to profiling the pharmacology of allosteric modulators. These compounds bind to the receptor at alternative sites to the orthosteric site (Figure 1A), are saturable in their efficacy, and generally require the endogenous agonist to be present to exert their effects. Allosteric ligands tend to be more selective for specific targets as they bind to the protein at unique sites and can provide highly specific tools for functional studies.^93^ In our studies here, we observed that an orthosteric antagonist and two NAMs that bind to different domains on the mGlu_7_ receptor protein retained efficacy in the presence of Elfn1. Exploration of a range of mGlu_7_ NAMs with different levels of blockade showed that these ligands could also block receptor activity, and a correlation analysis between the level of blockade in the presence of either empty vector control or Elfn1 showed a very high correlation across the range of NAMs examined. These results suggest that these ligands function similarly regardless of the presence of Elfn1. Our profiling of PAMs again showed a retention of activity; however, when performed in concentration–response format, maximal levels of potentiation for each PAM were reduced compared to the baseline in the presence of Elfn1, but all compounds demonstrated at least a 2-fold potentiation of receptor activity. We also determined that the PAM VU0422288, a compound we have used in slices and in vivo to increase mGlu_7_ activity,^55,63^ could potentiate responses in the presence of either l-AP4 or glutamate, the endogenous neurotransmitter, suggesting that there is no probe dependence between these agonists and VU0422288.

Our in vitro results suggested that the ligands profiled here should be active at synapses where Elfn1 is expressed. We tested this hypothesis using VU0422288 and showed that the compound retained activity in potentiating responses to l-AP4 at PYR → SST-IN synapses. Overall, our results suggest that these mGlu_7_ ligands will retain their pharmacology in native tissues regardless of the presence of Elfn1, providing confidence in the interpretation of pharmacological studies examining mGlu_7_ function in studies such as electrophysiology.

While the ligands tested here exhibit activity with or without Elfn1, it is intriguing to speculate on the utility of compounds that might be able to differentially modulate mGlu_7_ signaling in the presence versus absence of the Elfn proteins. The majority of studies performed thus far to study group III mGlu receptor/Elfn family member interactions have used shRNAs or knockout animals to reduce expression of one of the interacting partners. A small molecule or peptide tool that would target the specific modulation of the mGlu_7_/Elfn1 complex would allow for acute modulation (or lack of modulation) studies of SST-IN activity and have utility in understanding circuit-level regulation by SST-INs versus other types of interneurons that lack expression of Elfn1. Additionally, a compound that enhanced the interaction of mGlu_7_ and Elfn1 might serve as an intriguing therapeutic strategy in disorders such as epilepsy and/or ADHD where clinical loss-of-function data have already established a role for each protein.

As the majority of allosteric modulators for mGlu_7_ bind in the transmembrane domain and have been identified in cells without coexpression of Elfn1, new screening efforts may aid in the identification of compounds that affect the mGlu_7_/Elfn1 interaction. As Elfn1 binding is dependent upon glycosylation sites in the large extracellular Venus flytrap domain of mGlu_7_ ^8^ versus in the transmembrane domains of the receptor, the separation of binding sites may mean that new screening efforts performed in the presence of Elfns are needed to identify potential small molecules that can impact these interactions. It is also possible that “molecular glue”-like compounds^94^ or peptides may be able to disrupt or increase protein/protein interactions between mGlu_7_ and Elfn1 to provide opportunities for selective complex modulation. Additionally, the reduced potentiation observed in the presence of PAMs suggests that PAMs with weak efficacy may provide a mechanism to differentiate mGlu_7_ activity in the presence or absence of Elfn1.

## Methods

### Drugs

l-AP4 was purchased from Hello Bio (Princeton, NJ). l-SOP, LY341495, and XAP044 were purchased from Tocris (Bristol, U.K.). NAMs (ADX71743, VU6010608, VU6010953, VU6019281, VU6019282, VU6018226, VU6018229, VU6017743) and PAMs (VU0422288, VU0155094, VU6005649, VU6027459) were previously synthesized in-house as previously described.^50,51,55,95^

### DNA Construction

A carboxy-terminally tagged Elfn1-Myc construct^36^ was constructed by amplifying a mouse Elfn1 cDNA clone (Clone ID 6811341, Open Biosystems) and subcloning into pcDNA3 at the *Bam*HI/*Eco*RI sites. HEK293A polyclonal cells stably expressing either Elfn1 or empty vector were passaged in media (Dulbecco’s modified Eagle medium (DMEM), supplemented with 10% fetal bovine serum (FBS), 20 mM *N*-(2-hydroxyethyl)­piperazine-*N*′-ethanesulfonic acid (HEPES), 1 mM Na pyruvate, 2 mM Glutamax, 0.1 mM nonessential amino acids, 1× antibiotic/antimycotic) under G418 (700 μg/mL) selection to maintain expression.

### Cell Lines and Cell Culture

HEK293A cells stably expressing mouse Elfn1 or pcDNA3 were maintained in growth medium containing 90% Dulbecco’s Modified Eagle Media (DMEM), 10% fetal bovine serum (FBS), 100 units/mL penicillin/streptomycin, 20 mM HEPES, 1 mM sodium pyruvate, 2 mM l-glutamine, 1× nonessential amino acids, and 700 μg/mL G418 sulfate. HEK mGlu_7_/GIRK cells were maintained in a growth medium with 90% DMEM/F12, 10% dialyzed FBS, 100 units/mL penicillin/streptomycin, 20 mM HEPES, 1 mM sodium pyruvate, 2 mM l-glutamine, 1× nonessential amino acids, 700 μg/mL G418 sulfate, and 600 ng/mL puromycin. All cell culture reagents were purchased from Invitrogen (Carlsbad, CA).

### Thallium Flux Assay

Thallium flux assays were performed as previously described.^96^ A mixture of HEK293A mGlu_7_/GIRK cells and pcDNA3 or Elfn1-expressing HEK293A cells (5000/15,000 cells/20 μL/well for agonist and PAM experiments; 7500/15,000 cells/20 μL/well for NAM experiments) was seeded in 384-well, amine-coated assay plates (Greiner Bio-One, Monroe, NC). Cells were incubated overnight in a humidified 5% CO_2_ cell culture incubator at 37 °C. The cell culture medium was replaced with 20 μL/well of a dye loading solution containing the assay buffer (Hanks balanced salt solution plus 20 mM HEPES, pH 7.3), and 1.2 μM solution of the thallium-sensitive dye Thallos-AM (ION biosciences, Marcos, TX), prepared as a DMSO stock and mixed first in a 1:1 ratio with (w/v) Pluronic F-127 (Sigma-Aldrich, St. Louis, MO). Following a 1 h incubation at room temperature, the dye loading solution was replaced with 20 μL/well assay buffer and the plates were loaded into a Hamamatsu FDSS 7000 (Bridgewater, NJ). Data were acquired at 1 Hz (excitation 470 ± 20 nm, emission 540 ± 30 nm) for 10 s, followed by the addition of 20 μL/well of 10 μM NAM/PAM/DMSO, followed by an additional 4 min of data collection. At the 4 min mark, 10 μL/well of a thallium stimulus buffer (125 mM NaHCO_3_, 1.8 mM CaSO_4_, 1 mM MgSO_4_, 5 mM glucose, 12 mM Tl_2_SO_4_, 10 mM HEPES pH 7.4) along with serial dilution of glutamate or l-AP4 (2×) was added and data collection continued for an additional 2 min. Thallium flux data were analyzed by first dividing each point in each wave by the first point in that wave and then subtracting the average of the vehicle control response. The slopes of these vehicle control-subtracted waves were calculated over ten data points beginning 2 s after thallium stimulus addition and fit to a three-parameter logistic equation in GraphPad Prism (La Jolla, CA). In most cases, the final data were normalized to the response of the mGlu_7_/pcDNA3 with vehicle and 5 mM l-AP4 unless otherwise noted. Potency (pEC_50_) and maximum responses (*E*_max_) induced by compounds were determined using a four-parameter logistical equation in Prism. Basal activity was calculated with data points at the three lowest test concentrations. Maximum activity was calculated with data points at two highest concentrations. All experiments were replicated at least three times in duplicate or triplicate.

### Ex Vivo Electrophysiology

Transgenic mice (both male and female) expressing tdTomato fluorescent protein in SST-INs were used in this study. They were generated by crossing female homozygous SST-Cre (Sst^tm2.1(cre)Zjh^/J, Stock #: 013044, Jackson Laboratories) with male homozygous Ai9 mice (B6;Cg-Gt­(Rosa)­26Sor^tm9(CAG‑tdTomato)Hze^/J, Stock #: 007909, Jackson Laboratories). All animals were group housed with food and water available ad libitum, kept under a 12 h light/dark cycle with lights on from 6:00 AM to 6:00 PM, and were used for experiments during the light phase. All of the experimental procedures were approved by the Vanderbilt University Animal Care and Use committee and followed the guidelines set forth by the Guide for the Care and Use of Laboratory Animals. 7–12-week-old mice were anesthetized with isofluorane, and the brains were quickly removed and submerged in ice-cold NMDG-based cutting/recovery solution (in mM: 93 NMDG, 2.5 KCl, 1.2 NaH_2_PO_4_, 30 NaHCO_3_, 20 HEPES, 25 d-glucose, 5 sodium ascorbate, 2 thiourea, 3 sodium pyruvate, 10 MgSO_4_, 0.5 CaCl_2_, pH 7.3–7.4, 300–310 mOsm). Coronal slices containing the prefrontal cortex (FPC) were cut at 280 μm using a Leica VT1200S microtome (Leica Biosystems Inc.). Slices were transferred to a recovery chamber containing the NMDG-based solution for 8 min at 32 °C, and then to a room-temperature holding chamber for at least 1 h containing artificial cerebro-spinal fluid (ACSF) (in mM: 119 NaCl, 1.25 NaH_2_PO_4_, 2.5 KCl, 11 d-glucose, 26 NaHCO_3_, 2.5 CaCl_2_, 1.3 MgCl_2_) supplemented with 600 μM sodium ascorbate for slice viability. All buffers were continuously bubbled with 95% O_2_/5% CO_2_. Subsequently, the slice was transferred to a 32 °C submersion recording chamber mounted on the stage of an Olympus BX50WI upright microscope (Olympus, NY), where the slice was perfused with ACSF at a rate of 2 mL/min. Whole cell recordings were made from visually identified, fluorescence-labeled SST-INs in layer V of PFC slices under the upright microscope. Recording electrodes were prepared from borosilicate glass (Sutter Instruments) using a Narishige puller (model PP-830; Narishige International) and had a resistance of 3–4 MΩ when filled the following electrode solution (in mM): 125 K-gluconate, 4 NaCl, 1.5 KCl, 10 HEPES, 4 ATP-Mg, 0.3 GTP-Na, and 10 phosphocreatine (Tirs), with pH 7.3–7.4 and osmolality ∼ 290 mOsm. Only SST-INs exhibiting rebound spiking or regular spiking in response to hyperpolarizing or depolarizing current steps under current-clamp conditions were selected for further voltage-clamp studies. Excitatory postsynaptic currents (EPSCs) were recorded from layer V SST-INs at a holding potential of −80 mV and evoked by electrical stimulation (100 μs duration, applied every 15 s) through a concentric bipolar stimulating electrode placed around 100–150 μm from the recorded cell. The holding potential of −80 mV under voltage clamp is closed to the calculated reversal potential of Cl^–^ and thus the EPSCs recorded in the present studies had minimal GABA_A_-mediated component. Electrical stimulation applied near the recorded cell in the cortex presumably activates the local neurons preferentially. However, it is impossible to rule out the possibility of stimulating long-range synaptic inputs from other cortical and subcortical areas. The electrophysiological signal was amplified and low-pass filtered at 1 kHz using an Axon Multiclamp 700B amplifier (Molecular Devices, Sunnyvale, CA) and digitized at 20 kHz and acquired using a Clampex10/DigiData1440A system (Molecular Devices). All drugs were bath applied. Data were analyzed using Clampfit 10 (Molecular Devices), Excel (Microsoft), and Prism (GraphPad Software), and presented as mean ± SEM. Statistical analysis was performed using two-tailed paired *t* test, unpaired *t* test, or repeated measures (RM) one-way ANOVA with post hoc Dunnett’s test, as appropriate.

### Safety

Thallium was collected and disposed of according to the protocols of the Vanderbilt Department of Environmental Health and Safety.

## Supplementary Material



## Notes

Additional figures and tables including basal values for a range of mGlu_7_ NAMs, effect of NAMs on l-AP4-mediated responses, effect of PAMs on mGlu_7_ responses, pEC_50_ and maximal response values for l-AP4 with a series of mGlu_7_ PAMs, and pEC_50_ and maximal response values for mGlu_7_ PAMs, all in the presence and absence of Elfn1 (PDF)

## Abbreviations

GPCR: G-protein-coupled receptors
mGlu: metabotropic glutamate receptors
CNS: central nervous system
GABA: γ-aminobutyric acid
ELFN1 and ELFN2: extracellular leucine-rich repeat and fibronectin type III domain containing 1 and 2
PAM: positive allosteric modulators
NAM: negative allosteric modulators
SST-INs: interneurons express somatostatin
PV-INs: parvalbumin-positive interneurons
ADHD: attention-deficit/hyperactivity disorder
l-AP4: l-2-amino-4-phosphonobutyric acid
GIRK: G protein inwardly rectifying potassium
eEPSCs: evoked excitatory postsynaptic currents
PYR: pyramidal cell
