# Supplementary material for: Profiling the Impact of mGlu7/Elfn1 Protein Interactions on the Pharmacology of mGlu7 Allosteric Modulators
Source: ACS Chem Neurosci. 2025 Jul 21;16(15):2872–86. doi: 10.1021/acschemneuro.5c00174 (PMC12333015; doi:10.1021/acschemneuro.5c00174)
Supplement: Supplementary file 1 [file cn5c00174_si_001.pdf]

Supporting Information

Profiling the Impact of mGlu$_7$/Elfn1 Protein Interactions on the Pharmacology of mGlu$_7$ Allosteric Modulators

Xia Lei[1], Zixiu Xiang[1], Alice L. Rodriguez[1], Margaret L. Wilson[1], Colleen M. Niswender[1,2,3,4,*]

[1]Department of Pharmacology and Warren Center for Neuroscience Drug Discovery, Vanderbilt University School of Medicine, Nashville, TN 37232

[2]Vanderbilt Institute of Chemical Biology, Vanderbilt University School of Medicine, Nashville, TN 37232

[3]Vanderbilt Brain Institute, Vanderbilt University School of Medicine, Nashville, TN 37232

[4]Vanderbilt Kennedy Center, Vanderbilt University Medical Center, Nashville, TN 37232

*Corresponding Author colleen.niswender@vanderbilt.edu

Supplemental Figures

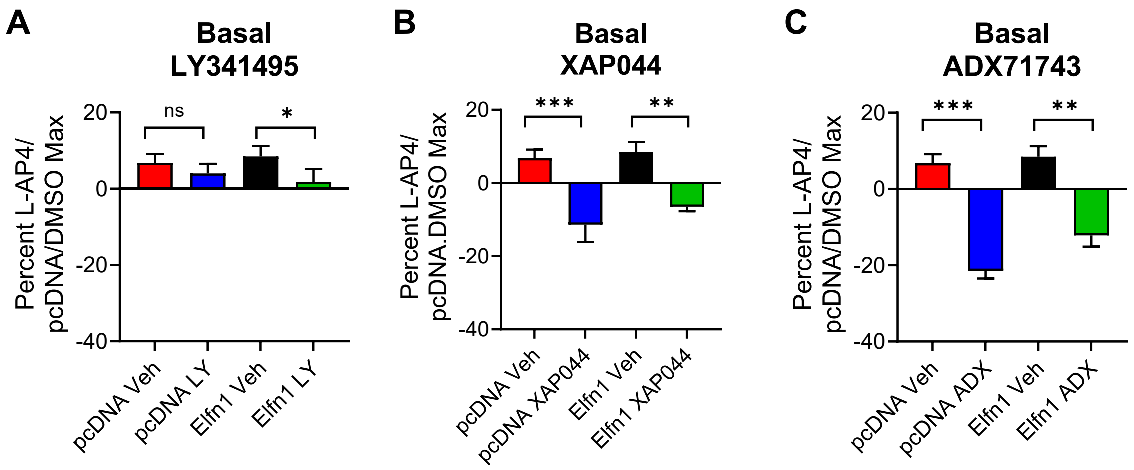

Figure S1. Basal values are significantly reduced by LY341495 in the presence of Elfn1 and both XAP044 and ADX71743 exhibit inverse agonist activity regardless of the presence of Elfn1. Data refer to curves in Figure 5A, E, and I. Red = pcDNA3 + DMSO, blue = pcDNA3 + antagonist, black = Elfn1 + DMSO, green = Elfn1 + antagonist. Basal effects quantified by averaging the response of the three lowest concentrations of L-AP4 for each condition. Unpaired t-tests between vehicle and antagonist conditions, *p<0.05; **p<0.01; ****p<0.0001; ns = not significant. Data represent three experiments performed in duplicate or triplicate and are shown as Mean ± SEM.

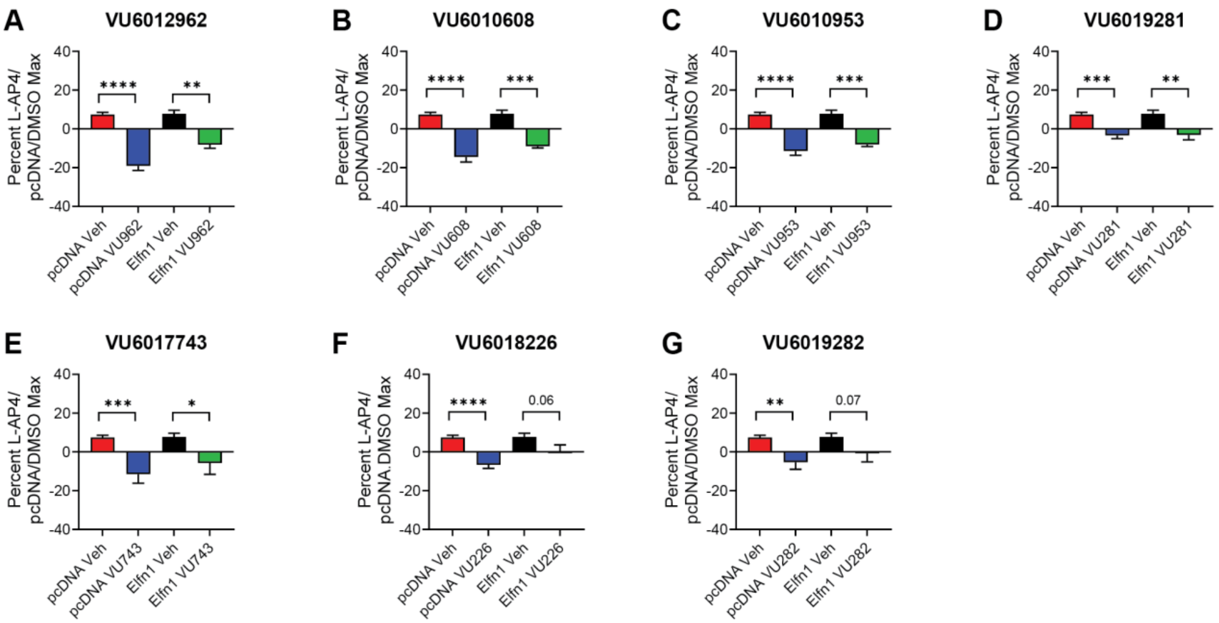

Figure S2. Basal values for a range of mGlu$_7$ NAMs are similar in the presence or absence of Elfn1. Data refer to curves in Figure 6. Red = pcDNA3 + DMSO, blue = pcDNA3 + antagonist, black = Elfn1 + DMSO, green = Elfn1 + antagonist. Basal effects quantified by averaging the response of the three lowest concentrations of L-AP4 for each condition. Unpaired t-tests between vehicle and antagonist conditions, *p<0.05; **p<0.01; ****p<0.0001; ns = not significant. Data represent three experiments performed in duplicate or triplicate and are shown as Mean ± SEM.

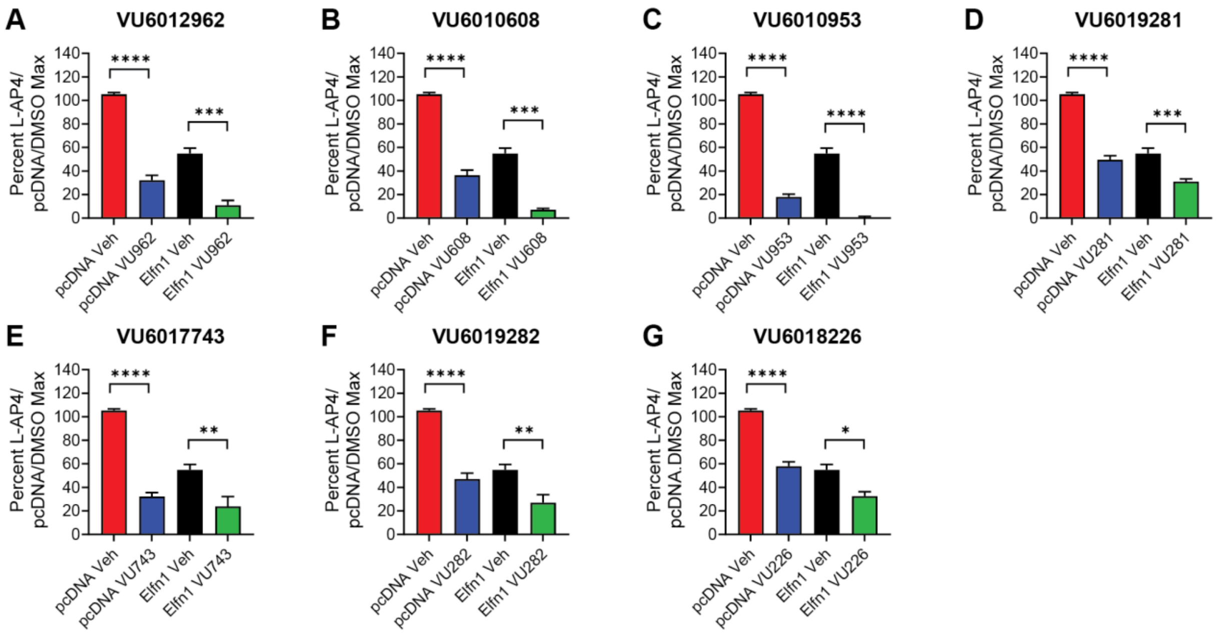

Figure S3. mGlu$_7$ NAMs block L-AP4-mediated responses in the presence or absence of Elfn1. Data refer to curves in Figure 6. Red = pcDNA3 + DMSO, blue = pcDNA3 + antagonist, black = Elfn1 + DMSO, green = Elfn1 + antagonist. Maximal effects were quantified by averaging the response of the two highest concentrations of L-AP4 for each condition. Unpaired t-tests between vehicle and antagonist conditions, *p<0.05; **p<0.01; ****p<0.0001; ns = not significant. Data represent three experiments performed in duplicate or triplicate and are shown as Mean ± SEM.

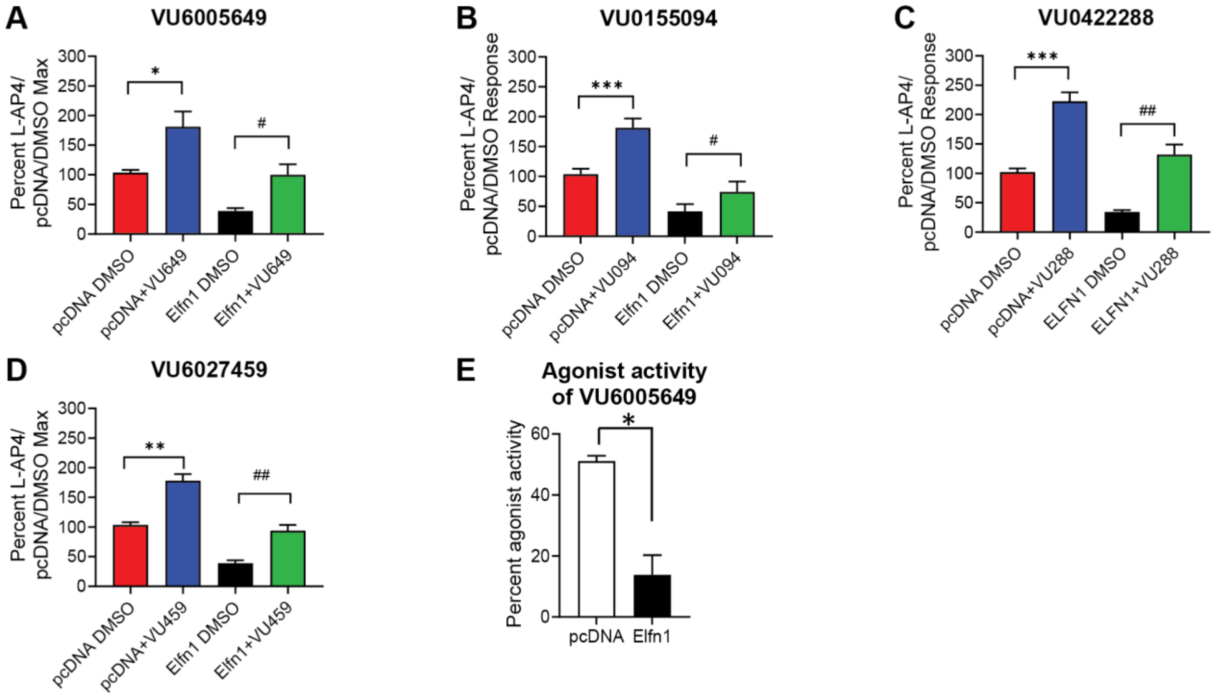

Figure S4. All examined PAMs potentiate maximal $mGlu_7$ responses in the presence or absence of Elfn1 and VU6005649 differentiates in agonist activity when Elfn1 is present. Data correspond to curve fits in Figure 8. **A.** VU6005649, **B.** VU0155094, **C.** VU0422288, **D.** VU6027459. Red = pcDNA3 + DMSO, blue = pcDNA3 + PAM black = Elfn1 + DMSO, green = Elfn1 + PAM. Maximal effects were quantified by averaging the response of the two highest concentrations of L-AP4 for each condition. Unpaired Student's t-test between vehicle and PAM conditions; *p<0.05; **p<0.01; ***p<0.001; ****p<0.0001; ns=not significant. **E.** Agonist activity for VU6005649 was calculated as the average of the bottom three concentrations of the L-AP4 curve. Unpaired Student's t-test between vehicle and PAM conditions, *p<0.05. Data represent three experiments performed in duplicate or triplicate and are shown as Mean ± SEM.

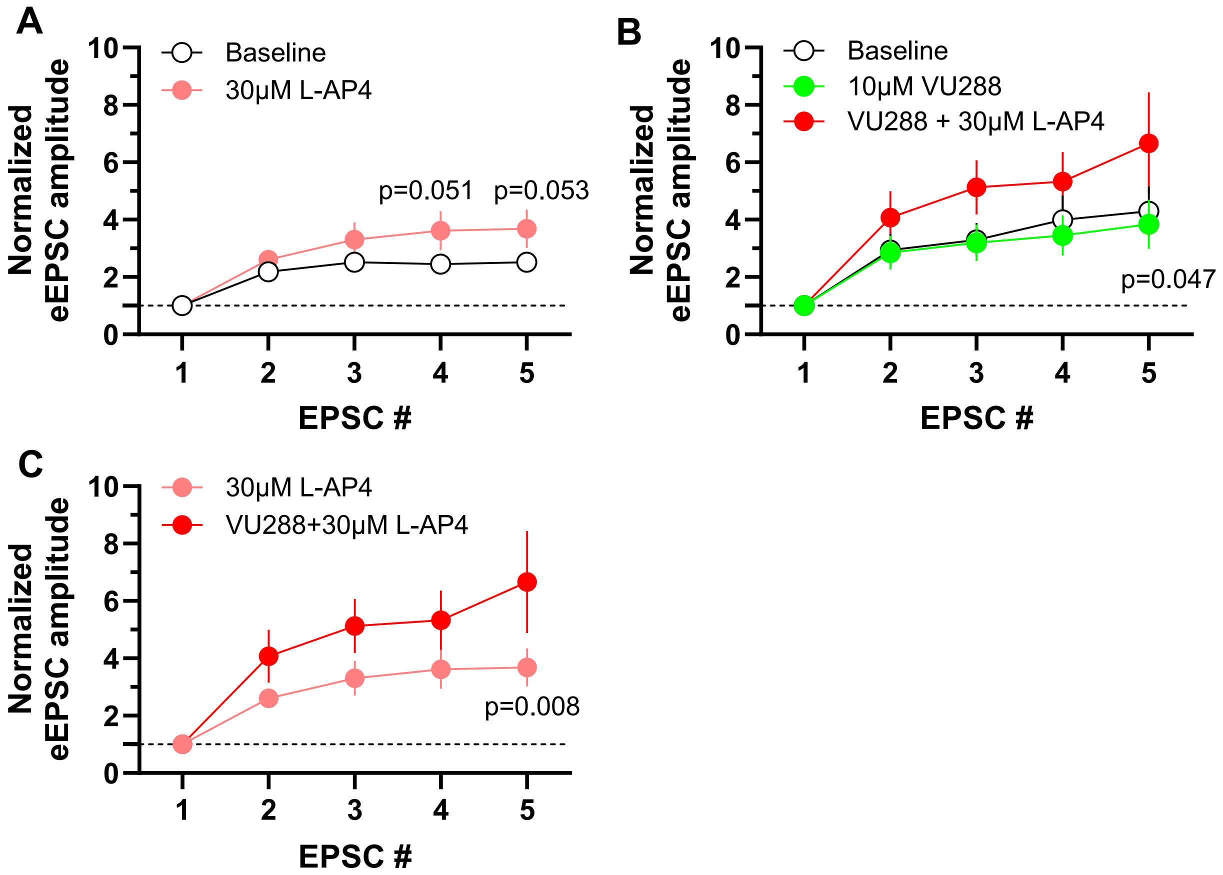

Figure S5. The effect of the PAM VU0422288 on short-term synaptic plasticity at SST-INs. (**A**) Normalized EPSC amplitude in response to 5-pulse stimuli at 50 Hz during baseline (*open circle*) and application of 30 μM L-AP4 (*light red)* (n = 8). RM two-way ANOVA, F (14, 56) = 7.962, p < 0.0001, with post hoc Tukey's test, p values indicate the comparison of normalized 4th and 5th EPSC amplitude in baseline and during application of L-AP4, respectively. (**B**) Normalized EPSC amplitude in response to 5-pulse stimuli at 50 Hz during baseline (*open circle*), application of 10 μM VU288 (*green*) and co-application of VU288 and 30 μM L-AP4 (*dark red)* (n = 5). RM two-way ANOVA, F (12, 48) = 6.713, p < 0.0001, with post hoc Tukey's test, p value indicates the comparison of the normalized 5th EPSC amplitude during application of 10 μM VU288 + 30 μM L-AP4 vs 10 μM VU288 alone. (**C**) Comparison of normalized 5-pulse stimulation evoked EPSC amplitude in response to co-application of 10 μM VU288 and 30 μM L-AP4 (*dark red*) vs application of 30 μM L-AP4 (*light red*). Data are from (A) and (B). RM two-way ANOVA, F (11, 44) = 6.075, p < 0.0001, with post hoc Tukey's test, p value indicates comparison of the normalized 5th EPSC amplitude in 10 μM VU288 + 30 μM L-AP4 group vs 30 μM L-AP4 group.

Supplemental Tables

Table S1. pEC_{50} and maximal response values for L-AP4 with a series of $mGlu_7$ PAMs in the presence and absence of Elfn1.

	Baseline Response ± SEM		pEC_{50} ± SEM		Maximal Response ± SEM	
	**pcDNA**	**Elfn1**	**pcDNA**	**Elfn1**	**pcDNA**	**Elfn1**
**DMSO**	-3.2±5.2	-5.1±1.4	3.62±0.03	3.70±0.03	103.5±4.6	38.8±5.0
**VU6005649**	60.4±5.8****	22.5±4.7$^{\#\#}$	3.97±0.06**	3.73±0.03$^{\#}$	181.1±25.7*	99.9±17.9$^{\#}$
**VU0155094**	6.4±2.3	1.0±1.7$^{\#}$	3.91±0.14*	3.64±0.06	171.4±4.4***	74.0±10.1$^{\#}$
**VU0422288**	11.4±5.2	0.7±5.3	3.86±0.07*	3.75±0.09	222.6±15.4***	131.9±17.1$^{\#\#}$
**VU6027459**	25.6±12.0	12.5±8.4	3.88±0.10**	3.48±0.17	178.1±11.3**	93.9±9.6$^{\#\#}$

Data are Mean ±SEM and refer to Figure 8 and represent 3-4 independent experiments performed in duplicate or triplicate. Student's t-tests between DMSO and PAM conditions for pcDNA (*) and Elfn1 ($^{\#}$), respectively. * $^{\text{or}\ \#}$ p<0.05, ** $^{\text{or}\ \#\#}$ p<0.01, *** or ****p<0.001.

Table S2. pEC_{50} and maximal response values for $mGlu_7$ PAMs in the presence and absence of Elfn1 at an EC_{20} concentration of L-AP4.

	**pcDNA**	**Elfn1**
**VU6005649**		
pEC_{50}	5.64±0.13	5.63±0.09
Maximal response	146.0±12.9	72.6±5.0***
Fold potentiation	6.1±0.7	2.6±0.3**
**VU0155094**		
pEC_{50}	5.45±0.06	5.55±0.05
Maximal response	138.0±5.2	66.3±3.6****
Fold potentiation	4.4±0.3	2.7±0.2***
**VU0422288**		
pEC_{50}	6.24±0.07	6.17±0.10
Maximal response	99.7±7.9	56.2 ±4.0****
Fold potentiation	2.9±0.2	2.3±0.2*
**VU6027459**		
pEC_{50}	6.00±0.10	5.79±0.13****
Maximal response	75.8±5.2	44.1±4.5**
Fold potentiation	3.0±0.4	2.0±0.2*

Data are Mean ±SEM and refer to Figure 9 and represent 5-12 independent experiments performed in duplicate or triplicate. Comparisons are performed between the pcDNA and Elfn1 conditions for pEC_{50}, maximal potentiation, and fold potentiation, *$p<0.05$, **$p<0.01$, ***$p<0.001$, ****$p<0.0001$.
